# Data on experimental investigation of Methyl Ester Sulphonate and nanopolystyrene for rheology improvement and filtration loss control of water-based drilling fluid

**DOI:** 10.1016/j.dib.2018.10.055

**Published:** 2018-10-24

**Authors:** Henry Elochukwu, Luke Kennedy S.L. Sia, Raoof Gholami, Mohamed A. Hamid

**Affiliations:** a Department of Petroleum Engineering, Curtin University Malaysia, Sarawak, Malaysia; b Department of Petroleum Engineering, Heriot-Watt Malaysia, Putrajaya, Malaysia

**Keywords:** Methyl Ester Sulphonate, Nanopolystyrene, Rheology, Fluid loss, Drilling fluid

## Abstract

Data presented in this article focused on the application of Methyl Ester Sulphonate (MES) surfactant and nanopolystyrene in water based drilling fluid. Data from rheology study using Bingham and Power law models showed that the synergy of MES and nanopolystyrene improved the formulated drilling fluid. Filtration study under LPLT and HPHT conditions showed that MES and nanopolystyrene drilling fluid reduced filtration loss by 50.7% at LPLT and 61.1% at HPHT conditions. These filtration data were validated by filter cake permeability and scanning electron microscope images.

**Specifications table**

**Table:** 

Subject area	Petroleum engineering
More specific subject area	Drilling/Drilling fluid
Type of data	Tables and Figures
How data was acquired	FANN viscometer, LPLP and HPHT filter press and Scanning Electronic Microscope (SEM).
Data format	Raw Data
Experimental factors	1. Dispersion of polystyrene nanofluid using ultra-sonificator 2. Preparation of samples for SEM measurement 3. Excessive drilling fluid are cleaned before filter cake thickness measurement.
Experimental features	
	Improvement of drilling fluid rheology and filtration properties by Methyl Ester Sulphonate (MES) and nanopolystyrene.
	Department of Petroleum Engineering, Curtin University, Malaysia.
Data source location	
	Data is made available with the article
Data accessibility	

**Value of data**

• Rheology result shows that methyl ester sulphonate an anionic surfactant affect water based drilling fluid properties: Yield Point (YP), Plastic Viscosity (PV) and flow behaviour characteristics.
• Synergy of methyl ester sulphonate and nanopolystyrene was effective to control fluid loss at low and high temperature environment.
• Filter cake formed after LPLT and HPHT filtration, showed a thin non-erodible low permeability filter cake which supports low fluid loss data obtained. This is further validated by Scanning Electron Microscope measurement.
• The overall result obtained from this work encourages the usage of environmentally friendly additives and environmental waste material as drilling fluid rheology and fluid loss control additives.

## 1 Data

Recently nanotechnology has seen many applications in oil and gas operations, from drilling to enhance oil recovery. Nanoparticles application in drilling fluid design has been reported in [1] and shown to have effect on rheology and filtration characteristics of drilling fluid. In particular, metallic/metal oxide nanoparticles have shown tremendous success especially filtration loss control and rheology improvement [2]. It is reported that metallic/metal oxide nanoparticles due to their magnetic and electrical properties has the potential to be used to design smart drilling fluids. However, the process of metallic/metal oxide nanoparticles manufacture are complex and expensive [3]. Meanwhile, non-metallic oxide nanoparticles have been reported to adversely affect rheology properties [4]. The dataset contained in this paper presents the investigation of Methyl Ester Sulphonate (MES) surfactant and nanopolystyrene to improve water based drilling fluid rheology and filtration loss control. Methyl Ester Sulphonate is an environmentally-friendly anionic surfactant while polystyrene is a waste material usually dumped at landfill site. The combination of both materials for application in oil and gas operation is an effort to reduce our environmental footprint.

## 2 Experimental design, materials and methods

Methodology for testing drilling fluid as recommended by American Petroleum Institute for water based drilling fluid (API 13B-1) was used for all rheology and filtration test.

### 2.1 Materials

Methyl Ester Sulphonate (MES) surfactant (279.7 g/mol), nano-polystyrene (25 nm) and bentonite. Other drilling fluid additives includes: sodium hydroxide (NaOH), barite and poly anionic cellulose (PAC).

### 2.2 Formulation of design drilling fluid

Base fluid was prepared by the addition of 15 g of bentonite to 350 cc of fresh water and stirred continuously for 15 minutes using 11,000 rpm mixer. 1 g of PAC was added and stirred for 5 minutes and 150 g of barite was added and stirred for another 10 minutes to prepare sample C. Sample D drilling fluid was prepared by dissolving 0.1 g of MES in deionize water and prepare 0.01 wt% nanopolystyrene with the MES solution. The final solution was ultrasonicated for 15 minutes to achieve good dispersion of nanopolystyrene in MES solution. Zeta potential measurement of dispersed nanopolystyrene in MES solution and polydispersity index are -41 mV and 0.15 respectively, which is indicative of stable nanofluid and well dispersed. 50 cc of prepared nanofluid was added to 300cc of sample C drilling fluid and mixed for 10 minutes. Two other drilling fluid was prepared: one with MES alone and base fluid (sample A) and the other is nanopolystyrene and base fluid (sample B). Table 1 shows the sample formulations and abbreviation for prepared drilling fluid sample.

**Table 1:** Drilling fluid samples and abbreviation.

Sample formulation	Abbreviation
Fresh water + 15 g Bentonite	Base fluid
Fresh water + 15 g Bentonite + 0.1 g MES	A
Fresh water + 15 g Bentonite + 0.01 wt% NP	B
Fresh water + 15 g Bentonite + 150 g Barite + 1 g PAC-L	C
Fresh water + 15 g Bentonite + 150 g Barite + 1 g PAC-L + 0.1 g MES + 0.01 wt% NP	D

### 2.3 Drilling fluid rheology

Rheology of designed drilling fluid was measured with a viscometer following API standard procedure recommended for water based drilling fluid (API 13B-1). Rheology measurement readings were carried out three times to ensure consistency and repeatability of the results. Rheological parameters of Bingham and Power law models including shear stress-shear rate relationship, plastic viscosity, yield point, gel strength, consistency and flow behavior index are determined. These rheology parameters were determined using the following:

**Table:** 

Power law: $\tau =K\gamma^{n}$	Shear stress = $\tau (\mathrm{lb}/100{\mathrm{ft}}^{2})$
Bingham model: τ=PV.γ+YP	Shear rate = $\gamma (s^{-1})$
$n=3.32\log\frac{\theta_{600}}{\theta_{300}}$	Flow behaviour index = n (Dimensionless)
$K=\frac{\theta_{600}}{1022^{n}}\;$	Consistency index = K $(\mathrm{lb}/100{\mathrm{ft}}^{2}.S^{n})$
	Plastic viscosity = PV(cP)
	Yield point = YP $(\mathrm{lb}/100{\mathrm{ft}}^{2})$
	600 dial reading = $\theta_{600}$
	300 dial reading = $\theta_{300}$

### 2.4 LPLT and HPHT filtration

Low Pressure Low Temperature (LPLT) filtration loss measurement conducted at operating condition of 100 psi and 24 °C. Filtrate volume was collected every 5 minutes for total filtration time of 30 minutes after which thickness of filter cake was measured. High Pressure High Temperature (HPHT) filtration loss was conducted at operating condition of differential pressure of 500 psi (600 psi of top regulator pressure and 100 psi of bottom backpressure) and temperature of 150 °C. Filtrate volume was monitored for every 5 minutes and accumulated filtrate volume after 30 minutes was recorded. Thickness of filter cake was also measured afterwards. Permeability of the filter cake was determined by the rate of filtration through the filter cake as described by Darcy׳s law. The Darcy׳s law calculation is as follows:

**Table:** 

$\frac{dV}{dt}=\frac{KA\Delta P}{\mu h}$	Rate of filtration = dV/dt
	Permeability of filter cake = K (mD)
	Differential pressure = ∆P (psi)
	Fluid viscosity = µ (cP)
$h=\frac{V_{f}}{A(\frac{f_{sc}}{f_{sm}}-1)}$	Thickness of filter cake = h (cm)
	Volume fraction of solids in filter cake = *f*_*sc*_
	Volume fraction of solids in drilling fluid = *f*_*sm*_
$V_{f}\;=\;A\sqrt{\frac{2K∆P}{\mu }\left(\frac{f_{\mathrm{sc}}}{f_{\mathrm{sm}}}-1\right)\times }\sqrt{t}$	Filtration time = t (min)
	Cross sectional area = A = 31.2 cm^2^

### 2.5 Scanning Electron Microscope (SEM) measurement of filter cake

Filter cake obtained after HPHT filtration was collected and dried in an oven at 100 °C for 24 hours. After which the samples were collected and carefully packaged to be sent for SEM measurement.

## 3 Result and discussion

**Fig. 1:**
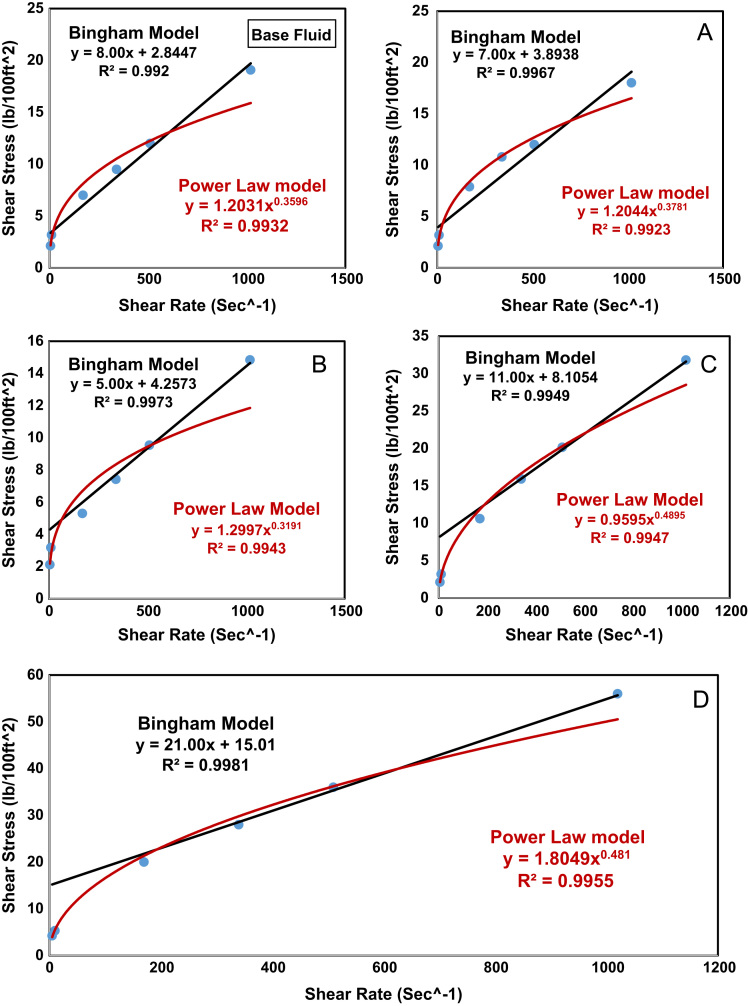
Shear rate-shear strain relationship plot for five prepared samples.

Bingham plastic model and power law model were used to evaluate rheology properties of five drilling fluid samples. Table 2 shows the rheological models of five drilling fluid samples. The plastic viscosity (PV) of sample D is relatively high compared to base fluid and other drilling fluid samples. However, it is sufficient for efficient drilling as the viscosity of drilling fluid increases it leads to higher penetration rate. Works of [5] concluded that plastic viscosity of nanoparticle drilling fluid in the range of 20–29 cP is considered sufficient to ensure circulation without inducing frictional pressure losses. Yield point (YP) obtained showed that addition of MES and nanopolystyrene aid the carrying capacity of drill cuttings. Sample D has the highest yield point, this is as a result of interaction of active additives which causes reduction in the electrostatic forces between drilling additives. The work of [6] reported yield point for nanoparticle drilling fluid in the range of 13.5 – 20.5 lb/100 ft^2^. Sample D yield point satisfies the operating requirement. Fig. 1 shows the shear rate and shear strain relationship, R^2^ values obtained indicate that PV and YP values obtained are good fit.

**Table 2:** Rheological models of five prepared drilling fluid samples.

Sample	Bingham plastic model			Power law model			10-min Gel strength (lb/100 ft^2^)
	PV	YP	R^2^	K	n	R^2^	
Base fluid	8.00	2.8447	0.9920	5.779	0.349	0.9932	8.00
A	7.00	3.8938	0.9967	5.314	0.349	0.9923	12.00
B	5.00	4.2573	0.9973	5.999	0.327	0.9943	15.00
C	11.00	8.1054	0.9949	4.604	0.489	0.9947	17.00
D	21.00	15.010	0.9981	6.386	0.477	0.9955	19.00

Power law model shows that all drilling fluid formulations showed a shear thinning behavior as indicated by flow behavior index (n) values. The values of flow behavior index are less than unit, which implies that as active additives such as MES and nanopolystyrene was included in the formulation of sample D, its plastic viscosity increased. Consistency index (K) has to do with hole cleaning capacity, as the values of K increases the annular viscosity increases. Sample D has the highest K value which implies that addition of MES and nanopolystyrene is effective for wellbore hole cleaning. Power law model R^2^ values is indicative that K and n values are a good fit. Gel strength describes behavior of drilling fluid when the pump is inactive and its ability to suspend drilling fluid particles from settling down. Excessive high gel strength will require high pump pressure to re-start circulation. Drilling fluid without nanoparticles, has 10-minute gel strength of 6–8 lb/100 ft^2^ [7]. This is in agreement with the values reported in this work.

**Fig. 2:**
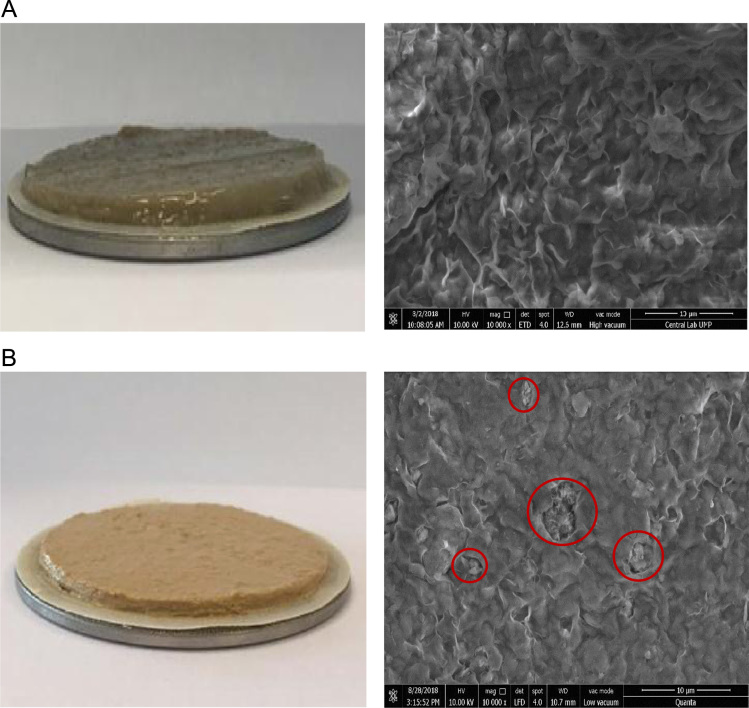
Scanning Electron Microscope of HPHT filter cake (a) Base fluid (b) sample D.

Filtration property of drilling fluid is dependent on the colloidal material additive included in the drilling fluid. Table 3 shows the filtration volume, volume change, filter cake thickness and permeability in filter cake at LPLT condition. The addition of nanopolystyrene to sample D resulted to a low filtrate volume loss with 50.7% reduction compared to base fluid. Also, thin filter cake thickness with low permeability supports the low filtration volume loss. At HPHT filtration condition presented in Table 4, the application of nanopolystyrene in sample D showed similar result of low filtrate volume with 61.1% reduction compared to base fluid. Filter cake thickness is low and permeability of the filter cake is the lowest in comparison to the other drilling fluid formulations. Scanning electron microscope was used to view the morphology of the filter cake of base fluid and sample D as shown in Fig. 2. Fig. 2(b) shows accumulation of nanopolystyrene in the filter cake indicating internal packing of nanopolystyrene which supports the low permeability of sample D. This type of internal packing is absent for base fluid filter cake as shown in Fig. 2(a). The texture of filter cake from Fig. 2(b) is different from Fig. 2(a) which is an indication that MES gave a better dispersion of additives to give a rigid filter cake.

**Table 3:** LPLT filtration volume, filter cake thickness and permeability of filter cake of five samples.

Sample	Filtrate volume (mL)	Filtrate volume change (%)	Filter cake thickness (/32 in.)	Filter cake permeability × 10^-2^ (mD)
Base fluid	15.2	–	3.0	8.5
A	15.0	−1.3	3.0	7.1
B	10.8	−29.0	2.0	5.2
C	9.0	_40.8	2.0	6.9
D	7.5	−50.7	2.0	1.1

**Table 4:** HPHT filtration volume, filter cake thickness and permeability of filter cake of five samples.

Sample	Filtrate volume (mL)	Filtrate volume change (%)	Filter cake thickness (/32 in.)	Filter cake permeability × 10^-4^ (mD)
Base fluid	18.0	–	3.5	3.2
A	18.0	+0.0	3.5	3.9
B	10.0	−44.4	3.0	2.7
C	8.0	−55.6	3.0	3.1
D	7.0	−61.1	2.0	2.2

It can be concluded, based on the data presented that methyl ester sulphonate (MES) which dispersed the drilling fluid additives improved the rheology of sample D formulation. Meanwhile, addition of nanopolystyrene effectively reduced drilling fluid filtrate loss and resulted to a thin non-erodible and low permeability filter cake. This filtration data was validated by SEM images of filter cake.
