# Supplementary material for: Data on experimental investigation of Methyl Ester Sulphonate and nanopolystyrene for rheology improvement and filtration loss control of water-based drilling fluid
Source: Data Brief. 2018 Oct 24;21:972–9. doi: 10.1016/j.dib.2018.10.055 (PMC6222072; doi:10.1016/j.dib.2018.10.055)
Supplement: Supplementary file 1 — Supplementary material [file mmc1.docx]

CDT 250 Jalan University,

98009 Miri,

Sarawak, Malaysia,

15th October, 2018.

The Editior,

Data-In-Brief.

AUTHOR DECLARATION OF NO CONFLICT OF INTEREST

We wish to draw the attention of the editor to the fact that there is no conflict of interest in this in this work submitted to Data-In-Brief. We have the permission of the University and the granting body to publish data from the work.

Thanks.

Henry Elochukwu.

Corresponding Author.
